# “Everyone needs to understand each other’s systems”: Stakeholder views on the acceptability and viability of a Pharmacist Independent Prescriber role in care homes for older people in the UK

**DOI:** 10.1111/hsc.12970

**Published:** 2020-03-02

**Authors:** Kathleen Lane, Christine Bond, David Wright, David P. Alldred, James Desborough, Richard Holland, Carmel Hughes, Fiona Poland

**Affiliations:** ^1^ University of East Anglia Norwich UK; ^2^ University of Aberdeen Aberdeen UK; ^3^ University of Leeds Leeds UK; ^4^ University of Leicester Leicester UK; ^5^ Queen’s University Belfast UK

**Keywords:** care homes, medicines management, older people, pharmaceutical care, prescribing, qualitative research

## Abstract

The role of an innovative Pharmacist Independent Prescriber (PIP) for care homes to optimise medications has not been examined. We explored stakeholders’ views on issues and barriers that the PIP might address to inform a service specification for the PIP intervention in older people's care homes. Focus groups (*n* = 72 participants) and semi‐structured interviews (*n* = 13) undertaken in 2015 across four sites in the United Kingdom captured the views of doctors, pharmacists, care‐home managers and staff, residents and relatives. Stakeholders identified their expectations of what service should be provided by PIPs, what might affect their support for the role, and barriers and enablers to providing the service. Transcripts were analysed using the Theoretical Domains Framework to identify key components, which were reviewed by stakeholders in 2016. A PIP service was envisaged offering benefits for residents, care homes and doctors but stakeholders raised challenges including agreement on areas where PIPs might prescribe, contextual barriers in chronic disease management, PIPs’ knowledge of older people's medicine, and implementation barriers in integrated team‐working and ensuring role clarity. Introducing a PIP was welcomed in principle but conditional on: a clearly defined PIP role communicated to stakeholders; collaboration across doctors, PIPs and care‐home staff; dialogue about developing the service with residents and relatives, based on trust and effective communication. To embed a PIP service within increasingly complex care‐homes provision, the overarching theme from this research was that everyone must “understand each other's systems”.

**What is known about this topic**

- Medication errors are common in care‐home environments.
- The current approach of pharmacists external to care‐home settings reviewing older residents’ medicines has demonstrated better medicines management but not improvement in clinical outcomes.
- To improve residents’ medicine‐related care, researchers recommend one person should take central responsibility for medicines management.

**What this paper adds**

- A prescribing pharmacist taking this central role is widely perceived as likely to reduce medication errors and improve residents’ medicine‐related care.
- For this innovation to work, everyone in care homes must understand each other's roles.
- Integrated team‐working, knowledge of older people's health and effective communication with residents and relatives were identified as essential to make the prescribing pharmacist role acceptable.

## 1 INTRODUCTION

It has been demonstrated that medicines management in residential settings for older people (hereafter referred to as “care homes”) in the United Kingdom (UK), should be improved (Care Quality Commission, 2010; Furniss et al., 2000; Kirthisingha, 2014). Providing a designated individual with overall continuing responsibility for the medicines management of individual care‐home residents might address identified shortcomings in medicine‐related care (Alldred et al., 2009). Pharmacist‐led medication reviews can achieve positive outcomes, such as reduced prescribing of inappropriate medications and resolving medication‐related problems (Alldred et al., 2009; Roberts et al., 2001; Zermansky et al., 2006). Furthermore, a model which relocates medication‐related decisions involving pharmacists more firmly within the context of care homes could improve resident‐related outcomes (Alldred, Kennedy, Hughes, Chen, & Miller, 2016; Patterson et al., 2014). Guidelines from the UK’s National Institute for Health and Care Excellence (NICE) recommend a holistic overview of medicines management (NICE, 2014), while a professional report proposes pharmacists having “overall responsibility for medicines and their use in care homes” and that a pharmacist and a family physician (in the UK called general practitioners [GPs]) share responsibility for medicines, “ensuring coordinated and consistently high standards of care” (Royal Pharmaceutical Society [RPS], 2016, p. 2). UK legislation (Department of Health, 2006) permits pharmacists to qualify as independent prescribers, able to diagnose and prescribe medicines, enabling them to implement changes following medication review, rather than recommending changes to a medical prescriber. Prescribing pharmacists may prescribe within their area of competence. For our study, the area of competence is “frail elderly residing in care homes”.

This paper reports an early‐stage qualitative study which explored stakeholders’ expectations and understandings of introducing a new service. This was part of the Care Homes Independent Pharmacist Prescribing Study (CHIPPS), a 5‐year, National Institute for Health Research‐funded research programme which is developing and evaluating the effectiveness and cost‐effectiveness of a novel Pharmacist Independent Prescriber (PIP) role to take overall clinical responsibility for managing repeat prescriptions in older people's care homes, aiming to optimise their medicines management (ordering, storage, prescribing, monitoring, administration) for an enhanced quality of life. It is following the iterative process recommended by the Medical Research Council for the development and evaluation of complex interventions, finishing with a definitive trial (Medical Research Council, 2000). The qualitative study was designed to inform how this service should be introduced, to mitigate potential barriers and deliver an acceptable service. The research question asked what components stakeholders would specify in a PIP service they deemed feasible and acceptable and what they considered barriers and enablers to implementing such a service. Stakeholders were accessed in four study sites: England (two), Scotland and Northern Ireland (one in each), all differing in demographic and cultural make‐up.

## 2 METHODS

### 2.1 Design

Qualitative methods drew on a phenomenological approach to enable us to explore less‐known ideas and priorities of stakeholders in the context of their experiences of care‐homes’ work organisation. Our approach drew on the Theoretical Domains Framework (TDF; Cane, O’Connor, & Michie, 2012) to systematise our search for contextual practices which stakeholders might judge as relevant to their actions within organisations and potentially affected by a PIP initiative. Framework analysis of the data was informed by the TDF, and was used to identify key components for a potential service specification and barriers and facilitators to changes in clinical practice which might accommodate a PIP service in care homes (Kirk, Sivertesen, Petersen, Nilsen, & Petersen, 2016; McGoldrick, Crawford, Brown, Groom, & Crowther, 2016).

### 2.2 Sampling

A purposive sampling approach to secure views of stakeholders with experience of living or working in or with care homes was taken to maximise the range of relevance of data collected to the research goals (Bryman, 2012). Stakeholders were GPs, pharmacists, care‐home managers, care‐home staff, care‐home residents and residents’ relatives, spanning urban and rural areas, single and multi‐GP practices and chain and family‐run care homes. GPs were contacted through research team and local professional networks. Pharmacists in primary care (linked to GP practices and providing care to care homes) were approached through health services organisations, while community pharmacists were contacted through local professional networks. Care homes were contacted through the national Care Quality Commission website and site‐specific regulatory bodies, local primary care and care‐home networks. A maximally varying sample was observed: care homes with or without nursing managers; with residents living with dementia; with both nursing and care residents and with diverse funding arrangements (local‐authority, private, mixed). Local network partners in each site emailed letters of invitation to each stakeholder group; this included a Participant Information Sheet and Expression of Interest Form. The latter invited interest to participate in a stakeholder‐specific focus group of up to eight participants. Potential participants were informed that, if unavailable for the focus group, they could ask to take part in an individual interview. Table 1 summarises participant numbers and types in each group.

**Table 1:** Numbers of participants per stakeholder group

Stakeholder group (abbrev.) and study sites involved^a^	Focus group (number of groups)	Interview	Total participants
Pharmacists (P)—all 4 UK sites	25 (4)	2	27
GPs (GP)—all 4 UK sites	24 (4)	5	29
Care‐home managers (CHM)—all 4 UK sites	3 (1)	3	6
Care‐home staff (CHS)—England (2) and Northern Ireland	6 (2)	3	9
Residents and Relatives (RR)—England and Scotland	7 Residents 7 Relatives = 14 (2)	0	14
Total	72	13	85

^a^ The four UK study sites: England (two), Scotland, Northern Ireland.

### 2.3 Data collection

Ethical approval was obtained from the NHS National Research Ethics Service on 10 April 2015 (REC reference, 15/YH/0172). A semi‐structured topic guide informed by the TDF for Behaviour Change was used to develop questions linked to constituent domains so as to identify expected characteristics, barriers and benefits of the proposed PIP role (Table 2). These included topics relating to knowledge, skills, environment, beliefs about consequences, practice features in the pharmacy context, older people in residential settings and primary‐care practice. We drew on the interdisciplinary research team's broad experience to identify both theory‐ and practice‐related data‐collection topics with context‐relevance. For example, questions exploring current medicines management aimed to tap stakeholders’ knowledge of scientific, procedural or environmental factors shaping care‐home practices, how a PIP service might work, and its potential benefits and risks. Questions eliciting stakeholder views on how the GP‐PIP partnership would work focused explicitly on social and professional roles and identity. Training‐related questions, while included on the topic guide, are reported in a separate paper.

**Table 2:** Topic guide for focus groups and interviews linked with domains in the theoretical domains framework

TDF domains (where applicable)	Question
	What is your role?
Knowledge Professional role Skills Environmental context	How are medicines managed at the moment in your experience for care‐home residents?
Knowledge Beliefs about capabilities Beliefs about consequences Skills	What might be the key ingredients of a PIP service?
Beliefs about capabilities Environmental context and resources	What organisational barriers could affect putting this into practice?
Social professional role and identity Social influences	What professional barriers could affect putting this into practice?
Intention Goals	What might be the solutions to these barriers?
Skills Behavioural regulation	What could we include in training pharmacists undertaking this role?
	Is there anything else important about this proposed service you want to mention?

Consent forms were distributed at each focus group and interview, where researchers also outlined their purpose and process. Information sheets were also available at each episode of data‐collection to help participants refresh their familiarity with the study. Focus groups for residents and relatives included the option for residents to have a carer for support (with two focus groups including carers supporting residents). We took care to communicate clearly with residents, respecting any needs for time to consider the process or answer questions; we reassured them that, as with all study participants, they were free not to respond or to leave the focus group, without giving a reason. Participants having telephone or Skype^©^ interviews received a consent form electronically, returning it to the research team prior to interview. All participants were given the opportunity to ask questions. Before commencing data‐collection, researchers double‐checked that participants understood that the discussion would be audio‐recorded. Focus groups lasted 60–90 min for GPs and pharmacists and 60 min for others, except for one residents’ and relatives’ group (32 min) and one with care‐home staff (21 min). Two researchers facilitated each focus group, one leading and the second note‐taking. Interviews, conducted by one researcher, lasted 25–35 min; three face‐to‐face and ten telephone or Skype^©^.

### 2.4 Analysis

All interviews were transcribed verbatim. Participants’ identities were anonymised and personally identifying references removed. Analysis began with repeated reading of the transcripts, to identify initial codes and themes from the data, then using the TDF‐informed questions (Table 2) to set up the framework analysis we had planned to use to identify key components relevant to our study design. Consensus about identified and emergent codes, themes and sub‐themes and their relationships was reached by two qualitative researchers engaging in an iterative process, supported by rigorous re‐reading of data. After the first round of analysis, an intervention‐development workshop was held at each site with invited local participants from mixed stakeholder groupings. The mixed‐group participants were presented with themes and topics from focus group and interview findings, as a stimulus from which they identified their specific issues with shortcomings and benefits in managing medicines in care homes and in light of what PIP‐related changes they foresaw. All of these were variously endorsed and elaborated in the stakeholder focus groups and interviews reported in the following findings.

**Table 3:** Characteristics of pharmacy professional participants

Sector	Prescribing pharmacists	Non‐prescribing pharmacists	Pharmacy technician	Total
Primary care	2	13		15
Community	1	9	1	11
Portfolio^a^	1	—		1
Total	4	22	1	27

^a^ Employed in a split role across primary care and community pharmacy.

## 3 FINDINGS

Thirteen stakeholder group‐specific focus groups (*n* = 72 participants) and 13 interviews (*n* = 13 participants) were held with GPs, pharmacists, pharmacy technician, care‐home managers, care‐home staff, residents and residents’ relatives in four study sites (Table 1). Of 27 pharmacy professional participants, 15 pharmacists worked in primary care, 10 in the community and one in both sectors; one pharmacy technician worked in the community (Table 3).

All focus group, interview and workshop stakeholders welcomed introducing a PIP into care homes in principle. Their reasons included viewing this new role as relevant for improving medicines management, benefiting residents, overcoming communication lapses between care home, GP practice and pharmacy, and providing medicines‐relevant communication between the GP, residents and their relatives. Nonetheless, stakeholders identified specific potential contextual and implementation barriers and facilitators for introducing an acceptable and viable PIP service in older people's care homes. Stakeholders strongly agreed that all parties must “understand each other's systems”, specifically in relation to:

- chronic disease management (*contextual*)
- knowledge of older people's medicine and care homes (*contextual*)
- clarity of a PIP’s role and responsibilities (*implementation*)
- integrated social and professional team‐working (*implementation*).

These are reflected in the organisation of findings now presented here.

### 3.1 Contextual barriers and facilitators

Chronic disease management and knowledge of older people's medicine and of care homes were seen to underpin an effective PIP service. Where lacking, these constituted contextual barriers. The barriers encompassed current knowledge and understanding, the potential need to reframe aspects of these, key organisations and stakeholder groups involved, cultural practices within care homes, governance and policies.

#### 3.1.1 Chronic disease management

All stakeholders emphatically identified chronic disease management as a core issue in managing medicines in care homes, emphasising monitoring and reviewing concerns. They considered a viable PIP service would depend on successfully addressing the “many points in the circuit of prescribing where it can go wrong” (GP15‐FG).^1^ They also highlighted a number of challenges to ensuring effective chronic disease management, such as working patterns which disrupted continuity of residents’ care, infrequent medication reviews, communication shortcomings in ordering medications, and minimal proactive oversight of medications within care homes.

GPs’ working patterns, together with the many care‐home staff involved with medicines, were seen to constrain effective chronic disease management and how residents’ medication needs were communicated. As a GP noted:

…one doctor’s introducing a new medication maybe for a short period … then the [care home] phone up and ask another doctor, ‘are we supposed to continue this?’. That’s maybe communication failures on our part but there’s more and more people becoming involved with each patient and …that can cause problems (GP5‐I)

All stakeholders prioritised regular, responsive medication reviews by PIPs as a way to address gaps in managing chronic disease and enhancing the safety of residents living with comorbidities. GPs’ onerous workload limited their capacity for “time‐consuming” procedures and “complexities” in reviewing and managing medication to “keep it right for this population” (GP8‐FG). Pharmacists acknowledged these limitations: they saw medicines dealt with as “an after‐thought” by time‐pressed doctors, whose care‐home visits were absorbed in treating acute problems, and argued that PIPs could do “more proactive work” (P10‐FG). For one GP, a PIP who helped “tie up all those loose ends” to ensure residents received appropriate medication and who liaised with GPs and community pharmacies “would be the answer to my prayers” (GP1‐FG).

GPs admitted that they found it “difficult managing all the complexity and the co‐morbidity” (GP6‐FG) and that “GPs don't understand” how care homes obtain and administer medicines (GP7‐FG). Care‐home systems incompatible with medicines supply were reported as impairing timely ordering and medicines supply. If a PIP oversaw and bridged gaps in these processes, the “mayhem” between care homes, pharmacies and GP practices, typified by “huge amount of telephone contact …with the [GP’s] receptionist over prescribing issues”, could be eliminated (GP7‐FG).

While no system appeared to ensure efficacy and efficiency in care‐home prescribing, a PIP model was seen to offer a means to address this—but only if well‐informed about older people's medicine and care homes.

#### 3.1.2 Knowledge of older people's medicine and care homes

Knowledge of older people's conditions and an appreciation of what it means to live in a care home were considered essential for the PIP. It was seen as vital that the PIP take into account the whole person, not the specific condition, recognise how care homes operate and understand practices and cultures within care homes.

Several GPs and pharmacists recommended PIPs be trained in geriatric medicine. One GP cautioned that pharmacists with little experience of older people might treat the condition as a “single system” (GP3‐I) rather than seeing the nuanced picture of older people's lives. Others recommended that PIPs learn about the impact of changing or starting medication on the overall health and quality of life of frail older people.

Pharmacists remarked that colleagues who were otherwise highly skilled might lack direct experience of care homes or be unaware of their protocols and regulatory frameworks. GPs, pharmacists and care‐home managers felt that PIPs must become conversant with mandatory guidelines and regulations governing medications in care homes. Knowledge of care‐homes’ organisational and cultural practices was also deemed necessary, one manager arguing that PIPs must “understand how care homes actually really function” (CHM4‐FG).

Stakeholders prioritised well‐developed communication skills to interact and share knowledge with residents, “particularly [those] with cognitive impairment” (P1‐FG). These skills included perceiving the whole person during medication reviews, such as noticing a resident's “hearing aid over on a table that they can't reach” (P5‐FG). One care‐home manager imagined a PIP initiating “a little chat” as a medications expert with residents (CHM2‐FG). For another manager, a PIP’s capacity to talk “with … not down to” residents was as important as pharmacological expertise (CHM3‐FG). GPs and pharmacists recommended that a PIP be sensitive to residents’ vulnerabilities regarding medications. This surfaced separately when a resident reported feeling “nervous” when “the appearance of a tablet changes”; she suggested a PIP could answer residents’ uncertainties about such changes (RR3‐FG).

### 3.2 Implementation barriers and facilitators

While stakeholders broadly accepted the proposed service, they questioned the PIP’s specific responsibilities and queried how the role would be understood and incorporated into the care‐home environment. Two implementation issues—clarity of the PIP’s role and responsibilities and integrated team‐working with the PIP—were seen to reflect the need to achieve effective multi‐professional team‐working.

#### 3.2.1 Clarity of PIP role and responsibilities

Stakeholders advocated clarity about the PIP role. Because GPs hold ultimate responsibility for patients’ healthcare, doctors argued that information be shared and monitored with the PIP, based upon effective, regular communication between them. Priorities for ensuring clarity were eliminating duplicate orders and preventing omissions in carrying out medication responsibilities. For care‐home staff, residents and relatives, a concrete and shared understanding of the PIP’s role would help to improve communication for the benefit of medicines management and residents themselves.

Stakeholders emphasised the need to understand each other's systems to increase the acceptability and viability of the PIP service. To promote such understanding, they recommended attaching PIPs to GP practices, thereby allowing PIPs to function successfully and benefit both care homes and GPs.

Care‐home managers raised potential barriers to the service from needing to respect residents’ traditional preference to discuss medical issues with their GP to providing extra training for care‐home staff about the new PIP role. The need to carefully consider residents’ and staff concerns was echoed by pharmacists and GPs. One GP believed that residents should be reassured that the PIP interacting with them had “one hand in the practice” (GP10‐FG). A pharmacist foresaw initial “apprehension” if all parties did not understand each other's roles or see benefits for residents (P9‐FG). As if exemplifying this point, a care‐home staff member initially queried why PIPs needed access to residents’ care records; on hearing fuller details about the PIP role, he revised his view of such access for supporting residents, with a colleague in his focus group adding, “all the medications will be reviewed … I think that would be nice” (CHS1‐FG). Such expressed doubts underlined that clear communication would be essential for maximising stakeholder understanding and acceptance of the role's remit.

A relative stressed their need to be kept aware of the new service “because often the residents can't pass on the information that the relatives would like to know” (RR2‐FG). Another relative saw a PIP meeting the significant need of transferring information from GPs to residents and of answering relatives’ questions more speedily than time‐pressed doctors, so that “everybody that needs to know is informed” (RR1‐FG).

Although no GP believed that the PIP’s role should include diagnosis, no GPs attached risk to a PIP monitoring and reviewing medicines or attending to time‐intensive matters such as synchronising residents’ prescriptions. GPs expressed confidence in pharmacists’ skills and applying these within the intervention, “looking at interactions and side‐effects” (GP3‐I) and bringing expertise “to guide us to things that we might have missed” (GP12‐FG).

Pharmacists unequivocally shared GPs’ reservations about diagnosing and endorsed the need for role clarity, including boundaries on expectations of the PIP’s tasks. One pharmacist acknowledged that “an ‘everything has to be done by a GP’‐culture” (P2‐I) among some care‐home staff could be changed through good models of working and clear role definitions. As one GP advised, “it's about framing your service so that actually people understand what benefit it's going to be for them” (GP13‐I). Stakeholders’ emphasis on role clarity and appropriate communication is further examined within the second implementation challenge: integrated team‐working.

#### 3.2.2 Integrated social and professional team‐working

Stakeholders believed that to embed the new service the PIP must establish communicative relationships with GP practices and care homes, from which shared understanding of roles, working co‐operatively rather than independently, developing trust and providing continuity of service could result. This would take time and depend on the PIP acquiring relevant experience and knowledge of the context of older residents’ health.

Many participants reflected on their positive experience of multi‐professional working when debating the benefits of integrating a PIP into a team; one cited an “effective working relationship” between GPs and pharmacists (GP6‐FG) and another valued pharmacists being linked to GP practices because “they're accountable to the GP” (P5‐FG). One GP urged PIPs be practice‐based because “nuances with managing the elderly [require] co‐operative working” (GP14‐FG) while another cautioned that a PIP acting independently of professionals involved with residents’ care risked being a “recipe for disaster for … looking after patients” (GP7‐FG).

Some GPs and care‐home managers conjectured that PIPs might educate care‐home staff to raise their awareness of medications. One doctor believed that this could “improve patients’ experience with tablets” (GP2‐FG). This resonated with residents’ desire to know about their medications:

…nobody [is there] to ask things about your medication, as the person giving you the drugs doesn’t have much knowledge, so they can’t explain (RR4‐FG)

Pharmacists also envisaged benefits for staff and residents if a PIP were an in‐house resource on medications. They suggested that a sensitive approach would increase the likelihood of collegial working if staff saw the PIP as part of a “care package” team for residents, rather than someone who might be “checking up on [staff]” (P6‐FG).

Stakeholders’ emphasis on clarity in the PIP role reinforced their need to establish whether and how this new role would add a distinctive and necessary new element to their multi‐professional working environment. Clearly defined team‐working for the benefit of residents would increase the new service's acceptability. Their attention to team‐working suggested specific ways that distinctive PIP contributions could be integrated to strengthen, not complicate, the complex collaborations on which care homes depend.

## 4 DISCUSSION

Our study explored stakeholders’ expectations of a feasible PIP service, the key components they specified for the role, the context of professional and multidisciplinary practices relating to care homes for the proposed service and the barriers and facilitators affecting its acceptability. Our TDF‐informed approach then enabled us to identify what components stakeholders deemed key and what contextual practices they saw as relevant to mitigate implementation barriers and to promote PIP feasibility and acceptability. This recognised the complexity of care‐home environments, with their many different players, organisational processes and systems and variations in resources providing care to frail older people.

Our comparative analysis revealed no specific inter‐country differences across the study sites. Stakeholders welcomed this novel role on many fronts, beginning with their expectation that the PIP would help to address chronic disease management challenges, in which medication reviews were made complex and time‐consuming by residents’ multiple co‐morbidities which complicated pharmacological interactions and side‐effects. GPs and most pharmacists emphasised needing to understand the complexities in managing the health of older care‐home residents and the impact of frailty and advancing age on their responses to drugs. Introducing a prescribing pharmacist in care homes was seen to offer specific potential benefits to residents, care homes and GPs.

Stakeholders believed that acceptability of the service would increase if the PIP offered the means to strengthen mechanisms to ensure efficient, effective prescribing in care homes. Repeated problems stemmed from no individual overseeing medication needs outside scheduled medicines management reviews. GPs and care‐home managers were left with the task of repairing treatment discontinuities when professional and organisational procedures contradicted each other and, echoing previous research, identified the absence of a “whole team” approach, no one with overall responsibility for medicines management and a lack of coordination (Barber et al., 2009).

Of primary importance to GPs was that a PIP initiative giving scope to the PIP to conduct medication reviews would save GPs time and work most largely given to solving acute problems when visiting care homes. They would not support the innovation if it would likely make their already burdensome workload more complex, as reported elsewhere for other well‐intended changes (Scott, Mannion, Davies, & Marshall, 2003). Potential benefits GPs identified were having better access to up‐to‐date knowledge on medication interactions and pharmaceutical guidelines. Although building multi‐professional relationships through the proposed PIP service would take time, GPs and pharmacists also saw these relationships as enhancing communication and trust around medications issues. Indeed, GPs predicated their welcome of the PIP initiative on establishing a trust‐based relationship which ensured mutually‐recognised remits and competencies (RPS, 2016).

We obtained comprehensive understanding from stakeholders of processes necessary to inform the intervention in order to maximise its chance of achieving a meaningful impact in practice (Davidoff, Dixon‐Woods, Leviton, & Michie, 2015). Central to the acceptability and viability of introducing a PIP was that everyone involved in implementing it “understand each other's systems”. This included recognising established organisational and cultural practices in care homes and primary care during implementation and ensuring good communication around related changes. A review of nursing‐home culture identified similar needs raised by introducing change within complex settings (Shier, Khodyakov, Cohen, Zimmerman, & Saliba, 2014). In this study, stakeholders’ knowledge of context and procedures revealed key ingredients to increase the viability of the PIP: both pharmacists and doctors argued that a PIP working in isolation from the GP practice or other health professionals in care homes might be a “disaster” for residents’ care. By contrast, they envisaged positive outcomes for a PIP embedded in collegial, communicative working with GP practices.

Both shared and distinct emphases surfaced across stakeholder groups. For example, all groups mentioned constraints on time‐pressed GPs in providing continuity of care for residents and so supported introducing a prescribing pharmacist to take overall responsibility for medicines management. Beliefs about a PIP’s capabilities and role were, however, expressed in different ways. Care‐home managers stressed the potential advantages of a PIP spending time with residents to explain medications, projecting both expert and social roles in the service, while residents and relatives saw in it a needed, helpful channel of timely communication and reassurance about medications. Such views reflect findings that care‐home residents are more satisfied when they are involved as full partners in their care and that healthcare support should reflect residents’ own perspectives (My Home Life, 2007; Social Care Institute for Excellence, 2013).

Integrated team‐working was another key component: stakeholders were likelier to consider the PIP role acceptable and viable if effective team‐working were embedded in its implementation, echoing research on healthcare change (Scott et al., 2003). Team‐working took several forms: care‐home managers observed that their staff could learn more about medication management from a PIP, care‐home staff expected a PIP could answer their questions about medicines while residents and relatives saw the PIP providing a reassuring sense of safety in medications. GPs strongly preferred that PIPs be integrated into their practice teams and acquire appropriate knowledge of older people's medicine to enable them to see residents holistically, not as detached systems. Pharmacists echoed these preferences, adding that PIPs could relieve some of the time‐pressures on GPs. Their views feed into the growing evidence that an extended role for pharmacists in care homes should help to reduce the burden placed on doctors (Chaplin, 2016), which in turn reflects the increasing variation in the UK’s primary‐care workforce. The widespread inter‐professional engagement with this research indicates the range of potential interest in the PIP role across the whole workforce.

A clearly defined PIP role was considered crucial. Residents and relatives made their likely acceptance of the new service conditional on having its purpose and elements carefully explained to them. These outcomes resonate with UK quality standards on involving families and carers in decisions about treatment and care in managing medicines in care homes (NICE, 2015). Explicit agreement on areas where PIPs might prescribe was essential for GPs and pharmacists. All participants emphasised that components of the PIP role be discernible at the micro‐level of individual actions, especially as experienced by residents and staff, and incorporated at the macro‐level of care home and GP practice organisation.

Although our findings are presented separately in contextual and implementation categories, these cannot be dissociated from each other in practice. Contextual factors framed how stakeholders envisaged implementation issues as making either more or less feasible the introduction of a PIP. For example, effective team‐working with the PIP, in GP practices and care homes (an implementation concern), depended in part upon the PIP acquiring appropriate knowledge of older people's medicine (a contextual issue). GPs saw working relationships as ideal if PIPs were practice‐based (implementation) and if chronic diseases were to be most successfully managed to benefit residents (contextual). Guidelines and existing research underscore that specifically addressing both context and implementation barriers is most likely to guarantee improved outcomes for older people in residential settings (Chaplin, 2016; NICE, 2015; RPS, 2016). As indicated above, stakeholders’ awareness of residents’ frailties regarding medication suggested that GPs, pharmacists, care‐home staff and relatives widely agreed on what specific knowledge, skills and environmental factors were critical to shaping the successful introduction and implementation of the PIP service. Linking multiple stakeholder views envisioning a proposed PIP innovation was the common belief that its acceptability and viability would depend on all stakeholders understanding each other's systems.

### 4.1 Limitations and strengths of this study

A limitation may have been the minimal representation of residents and relatives in intervention‐development workshops, perhaps because these were held on university premises outside their usual residence. There may have been bias in that participants were self‐selected and therefore perhaps had favourable expectations of the PIP role and may not have captured the views of individuals more likely to resist the innovation.

A particular strength was the independence of the qualitative researchers with no professional or clinical interest in this specific area of practice. Using the TDF to inform the approach for framing topics discussed with stakeholders strengthened the study by sensitising us to implementation issues relevant in the context of this proposed innovation. TDF use also facilitated stakeholders to reveal contextual and implementation barriers to defining, deploying and integrating a PIP role within existing services. It also drew comprehensively upon stakeholders’ experiences and awareness of context, social and pharmaceutical relationships.

## 5 CONCLUSIONS

For a new pharmaceutical care service to be effectively embedded, stakeholders’ views highlighted that securing the acceptability and viability of the PIP role would mean taking steps to ensure that all those involved in delivering and using it could “understand each other's systems”. Enabling such mutual understanding would address contextual and implementation barriers and be relevant to identifying feasible practices for addressing residents’ medications‐related safety and experience of medications. Current UK strategy aims to enhance care in care‐home settings partially through prescribing pharmacists. This paper demonstrates the widespread articulation by professional and lay stakeholders of the need for these pharmacists to take time to understand care‐home‐related systems they will operate within, before trying to enhance resident safety in better managing their medicines.

## CONFLICT OF INTEREST

Professor David Wright is in receipt of unrestricted education grants and undertakes consultancy work for Rosemont pharmaceuticals. No other authors have declared a conflict of interest.

## Supporting information

Supinfo

## ENDNOTE

^1^ Participants are indicated by stakeholder group abbreviation (see Table 1), individual ID and FG (focus group) or I (interview).
