# Supplementary material for: “Everyone needs to understand each other’s systems”: Stakeholder views on the acceptability and viability of a Pharmacist Independent Prescriber role in care homes for older people in the UK
Source: Health Soc Care Community. 2020 Mar 2;28(5):1479–87. doi: 10.1111/hsc.12970 (PMC7496840; doi:10.1111/hsc.12970)
Supplement: Supplementary file 1 — Supinfo [file HSC-28-1479-s001.docx]

Supplementary File: Further details on Recruitment and Confidentiality

*Recruitment*

Care-home managers in two UK regions acted as gate-keepers for recruiting residents and relatives to face-to-face focus groups. Recruitment was promoted by displaying posters in the care homes, giving residents a flyer and directing residents and relatives in resident groups to Participant Information Sheets. Recruitment documents emphasised that focus-group participation was voluntary and a decision either way would not affect the care they received. Expression of Interest Forms for residents and relatives, with stamped, addressed envelopes, were put in accessible places in each home, agreed with the manager.

*Confidentiality*

We addressed individually-specific needs for equitable informed consent, appropriate access to information and activities and support of participating residents and relatives. Relatives were eligible in their own right, whether their resident relatives had capacity or not.

Participants were told that whatever was reported in focus groups and interviews would remain confidential. It was stressed to focus-group participants that everything said in the group would remain in confidence. If participants disclosed anything which researchers considered to present a possible risk to themselves or others, this would be disclosed through the study sponsor to the relevant responsible authority. All participants were informed that transcriptions would be anonymised and exclude personally-identifiable features, each participant being assigned a study ID. No form of dissemination would identify participants by name, context or quotation.
